# Psychosocial interventions for people with amyotrophic lateral sclerosis and motor neuron disease and their caregivers: a scoping review

**DOI:** 10.1186/s12912-024-01721-6

**Published:** 2024-01-29

**Authors:** Juyeon Oh, Jiwon An, Kyongok Park, Youngok Park

**Affiliations:** 1 College of Nursing, Dankook University, 119 Dandae-Ro, Dongnam-Gu, Cheonan-Si, Chungcheongnam-Do 31116 South Korea; 2 Department of Nursing, Far East University, 76-32, Daehak-Gil, Gamgok-Myeon, , Eumseong-Gun, Chungcheongbuk-Do 27601 South Korea; 3 Department of Nursing, Gangneung-Wonju National University, 150, Namwon-Ro, Heungeop-Myeon, Wonju-Si, Gangwon-Do 26403 South Korea

**Keywords:** Amyotrophic lateral sclerosis, Caregiver, Motor neuron disease, Psychosocial intervention, Scoping review, Supportive care

## Abstract

**Background:**

As amyotrophic lateral sclerosis/motor neuron disease (ALS/MND) is a fatal progressive neurodegenerative disorder, patients experience severe impairments, with patients and family caregivers facing emotional distress and exhaustion. Several psychosocial interventions are aimed at providing tailored support for ALS/MND patients and caregivers. The aim of this study was to conduct a scoping review and present a comprehensive overview of psychosocial interventions designed for individuals and families affected by ALS/MND, while also pinpointing research gaps.

**Methods:**

This scoping review utilized Arksey and O'Malley's methodological framework to investigate psychosocial interventions designed for individuals with ALS/MND and their families. The study adhered to the PRISMA-ScR checklist for reporting.

**Results:**

A total of 27 articles describing 25 interventions met the inclusion criteria. The predominant interventions observed in the research encompassed education-related strategies, closely followed by behavior therapy, counseling, social support interventions, and psychotherapy interventions. Across the majority of the studies, findings indicated promising feasibility and acceptability of these interventions. Notably, a significant proportion of quantitative investigations yielded one or more statistically significant effects, while qualitative studies consistently reported favorable outcomes, including enhancements in well-being and heightened awareness of individual circumstances.

**Conclusions:**

Given the progressive and debilitating nature of this condition, coupled with the absence of a cure, the adoption of a psychosocial approach can prove beneficial for both ALS/MND patients and their families. However, high-quality RCTs with a large sample size are recommended to examine and confirm the effectiveness.

**Supplementary Information:**

The online version contains supplementary material available at 10.1186/s12912-024-01721-6.

## Background

Amyotrophic lateral sclerosis/motor neuron disease (ALS/MND), commonly known as Lou Gehrig's disease, is a progressive and devastating neurodegenerative disorder affecting motor neurons in the brain and spinal cord [1]. This disease results in the gradual degeneration of motor neurons, leading to muscle weakness, atrophy, and eventual paralysis [1]. As the disease advances, patients face increasingly severe consequences, including difficulties with basic tasks such as walking, talking, and swallowing. Respiratory muscles may also become compromised, necessitating ventilatory support. Ultimately, ALS/MND can lead to complete paralysis, making patients entirely dependent on caregivers for their daily needs [2]. The gradual loss of motor function for those with ALS/MND significantly impacts their quality of life. Findings from previous studies underscore the importance of tailored interventions to enhance the overall well-being of individuals with ALS/MND. Such interventions encompass multidisciplinary care, psychological support, and the incorporation of assistive technologies [3, 4]. Addressing the multidimensional aspects of well-being is crucial for providing comprehensive care and support for individuals facing this debilitating neurodegenerative disease.

Family members often become the primary caregivers for ALS/MND patients, assuming the responsibility for their daily care and well-being. The complex and progressive nature of ALS/MND can have a profound impact on both the physical and psychosocial well-being of both patients and their caregivers [5–7]. Caregivers may witness their loved ones' gradual deterioration, causing emotional distress. Additionally, the demands of caregiving can lead to physical and mental exhaustion, often resulting in caregiver burnout [8–10]. This impact extends to the caregiver's health and well-being, potentially jeopardizing their ability to provide effective care and affecting family dynamics [7]. Caregivers also face immense stress and burden as they take on caregiving responsibilities, navigate the healthcare system, and cope with the emotional toll of witnessing the progression of this disease [11, 12].

Psychosocial intervention refers to any attempt to provide solutions to the challenges individuals may encounter in terms of their psychological well‐being when interacting with any element of the social environment [13]. Psychosocial care includes a spectrum of services and therapies, spanning from educational interventions that systematically provide information to intensive one-on-one counseling sessions [14]. Psychosocial intervention has emerged as a therapeutic strategy designed to address the multifaceted challenges faced by ALS/MND patients and their caregivers [15–17]. Over the years, several psychosocial interventions have been developed and implemented to assist ALS/MND patients and their families to cope with the challenges posed by the disease [9, 18, 19]. Psychosocial interventions often combine multiple components to tailor the approach to the specific needs of each individual and their family. However, these studies were conducted with a heterogenous research design and content and used different measurements for testing effect, which make it difficult to obtain a comprehensive picture of psychosocial interventions both for people with ALS/MND and caregivers.

A prior scoping review focused on psychological interventions for people with MND [20] conducted a detailed and comprehensive analysis. However, it had a limited focus, centering on interventions only for patients and interventions primary with a psychological focus. Furthermore, several recent studies have been published since the completion of this scoping review. Therefore, the purpose of this scoping review is to provide a comprehensive overview of the existing literature on psychosocial interventions for people with ALS/MND and their caregivers, while also pinpointing research gaps. This review aims to inform healthcare practitioners, researchers, and policymakers about the current state of psychosocial interventions for ALS/MND, facilitating the development of more targeted and effective support programs for this vulnerable population.

## Method

A scoping review is a study used to summarize and evaluate research results prior to conducting a systematic literature review. It serves as a useful way to map the types, data, and key concepts of evidence in a specific topic or study, helping to identify differences between studies and find answers to a wide range of research questions [21]. This scoping review aims to provide a comprehensive overview of current studies on psychosocial interventions conducted for people with ALS/MND and their families. The study was conducted in accordance with the framework developed by Arksey and O'Malley [21], and the results were reported according to the Preferred Reporting Items for Systematic Reviews and Meta-Analysis extension for Scoping Reviews checklist (Additional File 1).

### Stage 1: identifying the research question

The research questions were as follows:

- What psychosocial interventions have been performed and studied for people with ALS/MND and their caregivers?
- What are the characteristics of the studies (research design, participants, outcome variables) and interventions (contents, methods)?
- What are the significant effects of the interventions?
- What are areas or aspects of a topic that have not yet been sufficiently explained?

### Stage 2. Identifying relevant studies

The data search encompassed journal papers published since 2000 and utilized four databases: PubMed, Cochrane Central, EMBASE, and CINAHL. Keywords such as (amyotrophic lateral sclerosis OR motor neuron disease OR Lou Gehrig’s disease) AND (psychoeducation OR counseling OR psychotherapy OR behavioral therapy OR cognitive therapy OR education* OR information* OR training* OR teaching OR social support* OR self-care OR self-management) AND (intervention OR trial OR program OR programme OR RCT) were combined using Boolean operators 'AND' and 'OR.' Table 1 and Additional File 2 present the details of the electronic. Furthermore, we examined references from reference lists and manually searched key journals; however, none were included as they duplicated the findings from the earlier electronic database searches.

**Table 1:** PubMed search strategy for psychosocial interventions for people with ALS/MND and their families

No	Search Query
#1	"Amyotrophic Lateral Sclerosis"[Mesh]
#2	"Amyotrophic Lateral Sclerosis"[TW] OR Gehrig*[TW] OR "Lou Gehrig*"[TW] OR "ALS"[TW]
#3	#1 OR #2
#4	"Psychotherapy"[Mesh] OR "Social Support"[Mesh] OR "Psychosocial Support Systems"[Mesh] OR "Patient Care Team"[Mesh] OR "Adaptation, Psychological"[Mesh] OR "Patient Education as Topic"[Mesh]
#5	Psychotherap*[TW] OR "Behavior Therap*"[TW] OR Psychoeducation*[TW] OR "Social Care"[TW] OR "Social Support*"[TW] OR "Psychosocial Support*"[TW] OR ((Patient*[TW] OR Multidisciplinary[TW] OR Interdisciplinary[TW]) AND ("Health Team*"[TW] OR "Health Care Team*"[TW] OR "Healthcare Team*"[TW] OR "care team*"[TW])) OR ((psycho*[TW] OR emotion*[TW] OR personal[TW]) AND (adjustment*[TW] OR adaptati*[TW])) OR (Coping[TW] AND (Behavi*[TW] OR skill*[TW] OR strateg*[TW])) OR "Patient Education*"[TW]
#6	#4 OR #5
#7	#3 AND #6
#8	#7 AND (("2000/01/01"[PDAT]: "3000/12/31"[PDAT]) AND (English[lang])

### Stage 3. Study selection

**Fig. 1:**
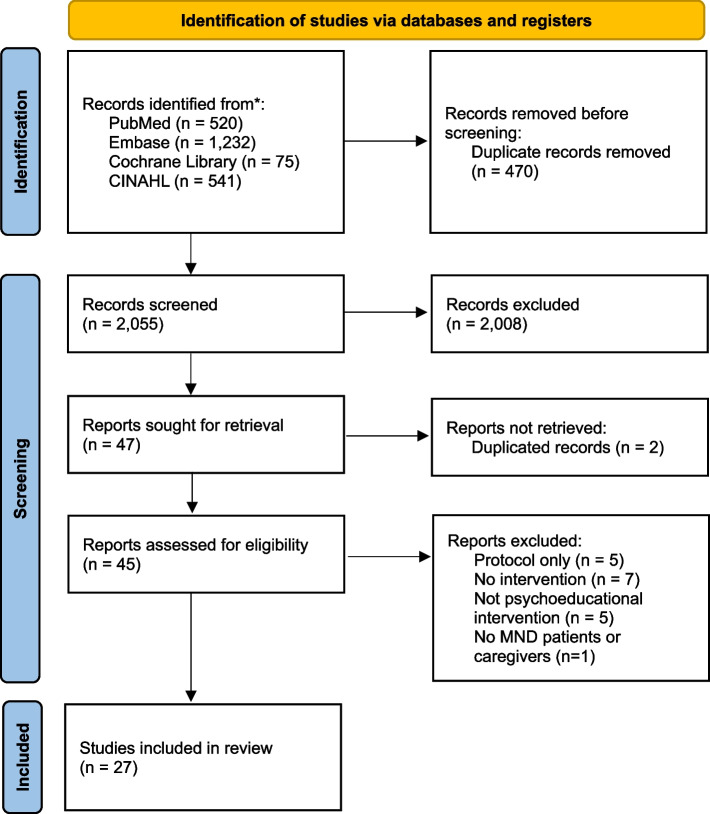
Preferred Reporting Item for Systematic Reviews and Meta-Analyses (PRISMA) Flow Diagram of Search Strategy

Duplicate articles were removed using the ‘find duplicates’ function in EndNote (Thomson Reuters; Carlsbad, CA, USA), and the selection criteria were applied to identify relevant studies. Articles were included if they met the following criteria: (1) a study of patients with ALS/MND or their families, (2) studies that provided interventions that fit the definition of psychosocial intervention [13, 14], (3) quantitative, qualitative, or mixed methods study design, (4) written in English, and (5) published since 2000. Academic conference posters, abstracts, protocols, commentaries, perspectives, or opinion papers were excluded (Fig. 1).

### Stage 4. Charting the data

The following data were extracted and summarized using Microsoft Excel:

- Publication information: author, year, country
- Research information: aim of the study, research design (mixed-method research, RCT, quasi-experiment, one-group pre-post study, case study, and qualitative studies), participants for data collection, measurements, key findings.
- Intervention information: contents (education, counseling, behavioral therapy, social support, or psychotherapy), target for the intervention (patients, caregivers, or both), delivery mode (individual or group-based / in-person or online or other methods), instructive approach (person who provided the intervention), average time per session, frequency, and duration

## Results

### Study selection

The initial search strategy identified 2,525 studies and after removing 470 duplicated studies, there remained 2,055 studies. After screening titles and abstracts, 2,008 were excluded, resulting in 47 potentially relevant studies. After excluding two duplicated studies, 45 studies were assessed for the eligibility with full-text, and finally 27 studies met the inclusion criteria (Fig. 1).

### Study characteristics

**Fig. 2:**
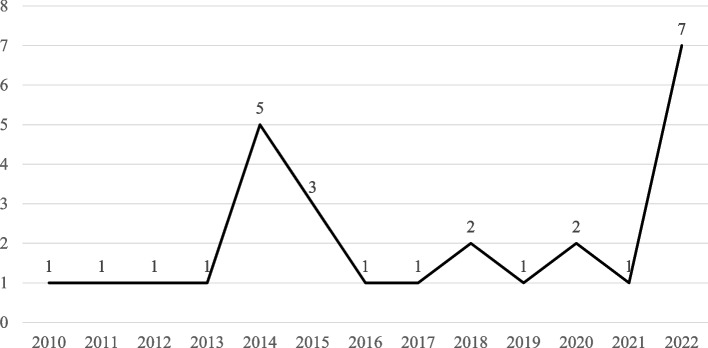
Number of studies published per year

A total of 27 studies were analyzed and general characteristics summarized, including authorship, publication year, research country, and research design in Table 2. As visualized in Fig. 2, one study [22] was published in 2010, 11 studies [7, 8, 23–29] were published between 2011 and 2015, seven studies [5, 6, 9, 10, 19, 30–33] were published between 2016 and 2020, and eight studies [18, 34–40] were published after 2021. Most studies were conducted in Italy (*n* = 9), Australia (*n* = 5), the Netherlands (*n* = 4), and USA (*n* = 3). Denmark, Iran, Japan, Russia, Spain, and Uruguay each published one study. In terms of study design, one group pre-post design [8, 22, 23, 25, 29, 32] and RCT [7, 9, 19, 24, 35, 38] were the most common with six studies, followed by five qualitative studies [6, 10, 18, 30, 31] and four quasi-experimental studies [5, 28, 34, 39], and three each for mixed methods [33, 36, 40] and case studies [26, 27, 37].

**Table 2:** Summary of Included Studies

**Authors (year)**	**Country**	**Research design**	**Aim of study**	**Participants for data collection**	**Contents**	**Target for intervention** - **delivery mode** **-instructive approach**	**Average time per session, frequency, duration**
Aoun et al. (2015) [23]	Australia	One group pre-post	To assess the acceptability, feasibility, and effectiveness of dignity therapy to reduce distress in people with MND and their family caregivers	27 patients (mean age: 64.3; female: 33.3%) 18 caregivers (mean age: 59.9; female:72%; spouse: 100%)	Counseling: *Dignity therapy*	Patients and caregivers (or only patients) - Individual; in person - Administered by a psychologist	average of 4 visits, average of 2 h per visit
Averill et al. (2013) [24]	USA	RCT	To examine effects and moderators of disclosure on psychological wellbeing in people with ALS	48 patients [E: 24, C:24] (mean age: 61.4 [E], 58.8 [C]; female: 37.5% [E], 25.0% [C])	Behavioral therapy: *Expressive disclosure*	Patients - Individual; giving assignments - Provided written instructions and telephone follow up	for 20 min a day for three days over a period of one week
Bentley et al. (2014) [8	Australia	One group pre-post	To assess the feasibility, acceptability, and potential effectiveness of dignity therapy for family carers of people with MND	18 caregivers (median age: 61; female: 72.2%; spouse: 100%)	Counseling: *Dignity therapy*	Patients and caregivers - Individual; in person - Administered by a therapist	Average of 3.75 (assisted by family carers), 4.41 (patients alone) sessions
Bentley et al. (2014) [25]	Australia	One group pre-post	To assess the feasibility, acceptability, and potential effectiveness of dignity therapy for people with MND	29 patients (range of age: 32–81; female: 31%; mean ALSFRS: 32.61)	Same as above*	Patients - Individual; in person, one participant with moderate speech impairment completed the intervention using video conferencing and email - Administered by a therapist	average of 4.4 sessions, average of 42 days
Bilenchi et al. (2022) [18]	Italy	Qualitative analysis	To describe the implementation of a structured psychoeducational intervention in ALS, identifying the needs of both patients and their caregivers	2 patients and 6 caregivers underwent an interview (5 patients and 13 caregivers joined the sessions)	Education, counseling, social support: *psychoeducational intervention*	Patients and caregivers - Group; in person - Administered by two psychologists	nine monthly sessions of 1 h and a half / 2 h each
Cipolletta et al. (2018) [30]	Italy	Qualitative analysis	To identify caregivers' needs, the prominent aspects of their experience, and to understand whether and how mutual support group intervention strategy might help them	12 caregivers (mean age: 62 [spouse], 41.5 [adult child]; female: 33.3% [spouse], 83.3% [adult child]; spouse: 50%)	Social support: *Mutual support group*	Caregivers - Group; in person - Coordinated by two facilitators	a total of 10 sessions of an hour and a half each
De Marchi et al. (2022) [34]	Italy	Quasi-experiment	To test the feasibility, safety, and tolerability of telehealth with a dedicated e-health Chatbot	46 patients [E: 26, C: 20] (mean age at onset: 57.2 [E], 59.8 [C]; female: 35% [E], 50% [C]; ALSFRS: 35.04 [E], 34.05 [C])	Education: *Chatbot webapp (dietary monitoring and nutritional recommendation)*	Patients - Individual; mHealth - Counseling through a chatbot	a total of 6 months. dietary intake was registered twice weekly, adjust monthly type of diet to be proposed
De Wit et al. (2020) [9]	The Netherlands	RCT	To evaluate a blended (face-to-face and online) psychosocial support program	148 dyads [E: 74, C: 74] caregivers (mean age: 61.8 [E], 61.3 [C]; female: 64.9% [E], 64.9% [C]) patients (mean age: 62.3 [E], 62.9 [C]; female: 35.1% [E], 36.5% [C]; mean ALSFRS: 31.7 [E], 31.0 [C])	Education, counseling, psychotherapy, social support: *Acceptance and Commitment Therapy*	Caregivers - Individual; in-person & online - Guided by a psychologist	A total of 8–12 weeks, 1 face-to-face contact (1 h), 6 online modules (1 h and 30 min per), 1 closing telephone calls (30 min)
De Wit et al. (2019) [31]	The Netherlands	Qualitative analysis	To gather insight into experiences with different components of a blended (face-to-face and online) psychosocial support program (program evaluation) and to discover what caregivers gained from following the program (mechanisms of impact)	23 caregivers (mean age: 59.6, female: 65%)	Same as above*	Caregivers - Individual; in-person & online - Guided by a psychologist	A total of 8–12 weeks, 1 face-to-face contact (1 h), 6 online modules (1 h and 30 min per), 1 closing telephone calls (30 min)
Díaz et al. (2016) [5]	Spain	Quasi-experiment	To evaluate the possible benefits of a psychological intervention based on a combination of CBT and counselling techniques	54 patients [E: 30, C: 24] (mean age: 60.6 [E], 66.2 [C]; female: 73.3% [E], 66.7% [C]; mean ALSFRS: 17.4 [E], 14.6 [C])	Education, counseling, behavioral therapy: *cognitive behavioral therapy combined with counselling techniques*	Patients and caregivers separately - Individual; in-person - Administered by health professionals	four semi-structured sessions, 1 h per session, 15–25 days between each session
Fateh et al. (2022) [35]	Iran	RCT	To investigate the effect of energy conservation techniques in patients with MND	28 patients [E: 14, C: 14] (mean age: 49.57 [E], 53.71 [C]; female: 50.0% [E], 50.0% [C])	Education, behavioral therapy: *Energy conservation program*	Patients - Not reported - Administered by an occupational therapist	3 weekly 1-h per session
García Pérez & Dapueto (2014) [26]	Uruguay	Case study	To describes a psychotherapy intervention in a patient in advanced stages of ALS	1 patient (age: 66, female)	Counseling: *psychotherapy using augmentative and alternative communication*	Patient - Individual; in-person - Administered by a psychotherapist	once a week, 1 h per session
Horne-Thompson & Bolger (2010) [22]	Australia	One group pre-post	To compare the effectiveness of a live music therapy session, recorded music, and silence, in reducing anxiety for patients with ALS/MND	21 patients (mean age: 61.7; female: 28.6%; mean ALSFRS: 22)	Behavioral therapy: *a live music therapy session, recorded music*	Patients - Individual; in-person - Administered by a music therapist	30 min each of the three study interventions: a live music therapy session, a recorded music intervention and a control intervention
Jikumaru (2011) [27]	Japan	Case study	To test the utility of the Psychotherapeutic Nursing in an advanced stage of the ALS patient	1 patient (age: mid-fifties, male)	Counseling: *psychotherapeutic Nursing*	Patient - Individual; in-person - Administered by a psychotherapist-nurse	29 sessions, once a week, 45–60 min per session
Kavanaugh et al. (2020) [32]	USA	One group pre-post	To test whether young carer participants in the YCare protocol show improved self-efficacy in care tasks after receiving the training; and whether youth participants can identify self-care goals and behaviors toward the development of self-management as caregivers	19 caregivers (range of age: 9–19, female: 31.58%, child of a patient: 78.95%)	Education, social support: *skills training and support program*	Caregivers - Group; in-person - Administered by health professionals	four modules, 50 min per module
Kleinbub et al. (2015) [28]	Italy	Quasi-experiment	To test the effect of hypnosis-based intervention to a broader ALS group, investigating the longitudinal, long-term effects of intervention on patients and their caregivers, and taking into account the impact on disease progression as well as the influence of individual aspects	30 patients [E: 15, C: 15] (mean age: 55.3 [E]; female: 53.3% [E]) 15 caregivers [E: 15] (female: 66.7%, spouse: 80.0%)	Psychotherapy: *a hypnosis treatment and self-hypnosis training*	Patients - Individual; in-person - Administered by a trained operator in Ericksonian hypnosis	4 weekly sessions, 60-75 min per session
Kolomeytseva et al. (2022) [36]	Russia	Mixed method	To determine the feasibility of a home-based music therapy protocol as an intervention to support respiratory and bulbar functions in early- and mid-stage ALS	8 patients (mean age: 58.1; female: 75.0%; range of ALSFRS: 31–42) 6 caregivers (female: 66.7%, spouse: 66.7%)	Behavioral therapy: *home-based music therapy*	Patients - Individual; in-person - Administered by a music therapist	twice weekly for six weeks
Madsen et al. (2019) [6]	Denmark	Qualitative analysis	To gain insight into experiences and reflections of persons with ALS and relatives concerning the peer group rehabilitation program “More Life–Less Illness.”	8 patients (age of 60–69: 50.0%; female: 25.0%) 10 caregivers (female: 90.0%; spouse: 80.0%)	Social support: *‘More life-Less illness’ (peer group rehabilitation)*	Patients and caregivers - Group; in-person - Facilitated by a program professional	6 sessions, monthly, half a day per session
Marconi et al. (2016) [10]	Italy	Qualitative analysis	To investigate the experience of a meditation training program tailored for people with ALS and their caregivers	26 patients (mean age: 61.9; mean ALSFRS: 29) 18 caregivers (mean age:57.8)	Psychotherapy: *meditation training program*	Patients and caregivers - Group; in-person - Conducted by two trainers	8 sessions, weekly
Oudman & Baert (2022) [37]	Netherlands	Case study	To describe the positive effects of eye movement desensitization and reprocessing (EMDR)	1 patient (age: 48, female)	Psychotherapy, behavioral: *EMDR and mediative behavioral therapy*	Patient - Individual; in-person - Conducted by psychologists	2 weeks of mediative treatment, 40 min of EMDR session
Pagnini et al. (2022) [38]	USA	RCT	To explore the impact of an ALS-specific online Langerian mindfulness training program on quality of life of ALS patients	38 patients [E: 19, C:19] (mean age: 63.9 [E], 62.3 [C]; female: 55.6% [E], 47.4% [C]) 22 caregivers (E:11, C:11) (mean age: 58.1 [E], 62.3 [C]; female: 44.4% [E], 72.7% [C])	Psychotherapy: *an ALS-specific online Langerian mindfulness training program*	Patients and caregivers - Individual [dyads or patient or caregiver alone]; online - Facilitated using email	5 weeks, daily exercises (mostly 2–3 min)
Palmieri et al. (2012) [39]	Italy	One group pre-post	To investigate the effect of hypnosis-based intervention on psychological and perceived physical wellbeing in patients and the indirect effect on caregivers	8 patients (mean age: 55; female: 50.0%; mean ALSFRS: 35) 8 caregivers (female: 62.5%, spouse: 87.5%)	Psychotherapy: *hypnosis intervention and self-hypnosis training*	Patients - Individual; in-person - Administered by a psychologist	four weekly sessions and self-hypnosis at least once everyday
Palmieri et al. (2021) [39]	Italy	Quasi-experiment	To compare two specific psychological interventions to reduce suffering and improve wellbeing in people with ALS, namely Rogerian supportive counseling and hypnosis-based intervention	36 patients [E: 21, C1:15, C2: 15 (from previous study)] (mean age: 65.35 [E], 65.8 [C1]; female: 38.1% [E], 20.0% [C1]; mean ALSFRS: 34.48 [E], 38.8 [C1])	Education, counseling: *empathy-based supportive counseling intervention*	Patients - Individual; in-person - Administered by a psychologist	four weekly sessions, 60 min per session
Raglio et al. (2016) [19]	Italy	RCT	To evaluate the possible efficacy of active music therapy mainly in psychological aspects of the disease, in particular, on anxiety, depression, and quality of life	30 patients [E: 15, C:15] (mean age: 62.9 [E], 65.1 [C1]; female: 53.3% [E], 60.0% [C1])	Behavioral therapy: *active music therapy*	Patients - Individual; in-person - Administered by a music therapist	12 sessions, three times a week, 30 min per session
Sharbafshaaer et al. (2022) [40]	Italy	Mixed-method	To explore the potential role of psychological support interventions for family caregivers of patients with ALS through resilience-oriented sessions of group therapy during the COVID-19 pandemic	12 caregivers [E: 6, C: 6] (mean age: 60.33 [E], 53.29 [C]; female: 66.7% [E], 57.1% [C]; spouse: 83.3% [E], 74.1% [C])	Counseling, social support: *Individual tele-consult and resilience-oriented sessions of group therapy*	Caregivers - Individual & group; online - Administered by a psychologist/psychotherapist	3 months, individual video-consults (1/month) & group therapy (2/month), 60 min per session
Ugalde et al. (2018) [33]	Australia	Mixed-method	To investigate the feasibility and acceptability of a therapeutic group intervention promoting self-care, problem-solving and mindfulness to informal caregivers of people with MND	13 caregivers (mean age: 57; female: 46%, spouse: 92%)	Education, counseling, social support: *Therapeutic group intervention*	Caregivers - Group; in-person - Conducted by two clinical psychologists	2.5 h, two sessions
van Groenestijn et al. (2015) [7]	the Netherlands	RCT	To compare the effects of CBT and usual care on quality of life in patients with ALS and their caregivers	15 patients [E: 10, C: 5] (mean age: 57.4 [E], 54.8 [C]; female: 40.0% [E], 40.0% [C]; mean ALSFRS: 41.0 [E], 40.0 [C]) 10 caregivers (mean age: 57.3 [E], 53.4 [C]; female: 70.0% [E], 60.0% [C]; spouse: 100.0 [E], 100.0 [C])	Education, counseling: *CBT sessions*	Patients and caregivers - Individual [dyads or patient or caregiver alone]; in-person - Conducted by three psychologists	Median of 3 sessions, 1 h per session

*ALS* Amyotrophic lateral sclerosis, *ALSFRS* ALS functional rating scale, *C* control group, *CBT* cognitive behavioral therapy, *E* experimental group, *MND* motor neuron disease, *RCT* randomized controlled trial

### Participant characteristics

The range of sample sizes used in data collection varied widely from one [26, 27, 37] to a maximum of 148 [9]. Most of the studies involved less than 50 participants, two studies [5, 38] 50–100 participants, one study [9] had over 100 participants. Eleven studies [5, 19, 22, 24–27, 34, 35, 37, 39] collected data from people with ALS/MND, six studies [8, 30–33, 40] from family members, and ten studies [6, 7, 9, 10, 18, 23, 28, 29, 36, 38] from both persons with ALS/MND and also family members.

### Intervention characteristics

**Fig. 3:**
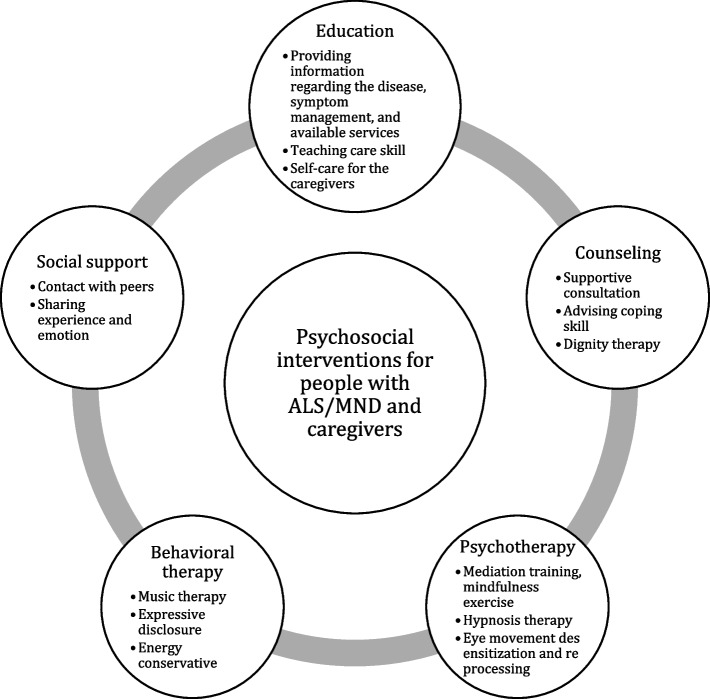
Psychosocial Interventions for People with ALS/MND and Caregivers

Content, targets, delivery mode, instructive approach, and frequencies of psychosocial interventions of the reviewed studies are summarized in Table 2. In terms of content, a diverse range was observed (Fig. 3). When an intervention included multiple components of a content category, each was counted individually. When interventions [8, 9, 25, 31] were published in two separate papers, we counted each intervention only once. Among the various interventions, education-related interventions were the most prevalent, totaling 9. This was followed by behavior therapy, counseling, and social support interventions, each comprising 7, and psychotherapy interventions, totaling 6. Education-related interventions included programs such as providing information regarding disease [39], skill training [32], teaching coping skills and problem solving [33], and nutrition education and counseling [34]. Behavior therapy interventions encompassed programs like expressive disclosure exercise [24] and music therapy [19, 22, 36], while counseling interventions included dignity therapy [8, 23] and supportive counseling [39]. Social support interventions included programs such as a mutual support group program [6, 30], and psychotherapy interventions featured treatment such as hypnosis treatment [28, 29], eye movement desensitization and reprocessing [37], and meditation [10].

The number of interventions provided to people with ALS/MND was 12 [19, 22, 24–29, 34, 35, 37, 39], while provided to family members was five [9, 30, 32, 33, 40], and including both persons with ALS/MND and family members was eight [5–8, 10, 18, 23, 38]. Eighteen interventions were provided individually and remaining interventions [6, 10, 18, 30, 32, 33, 40] were administered in a group setting. Most interventions were conducted in face-to-face settings. The remaining interventions included two delivered online [38, 40], one employing a blended approach (combining face-to-face contact and online modules) [9], and one utilizing a webapp-based chatbot [34]. Health professionals, such as psychologists, therapists, and nurses, predominantly led the majority of interventions. However, a subset of interventions was facilitated through alternative means, including email [38] or chatbots [34]. The duration of interventions per session varied, ranging from less than 10 min [38] to half a day [6]. The total intervention periods also exhibited diversity, spanning from one week [24] to nine months [18].

### Intervention outcomes

In the majority of the studies, the outcomes related to feasibility aspects, including retention, adherence, and tolerance, largely indicated the feasibility of the interventions. Dropout rates were various, but several studies were high at up to 48% [38], those dropouts were primarily attributable to factors such as the deterioration of patients' health, mobility limitations, and the time constraints faced by family members [9, 31]. The majority of the studies reported favorable acceptability results, such as high levels of participant satisfaction and perceived benefits. Nevertheless, a few studies identified specific limitations arising from challenges such as patients' communication difficulties, mobility issues, participants' prior knowledge levels, and the heterogeneity in disease severity [18, 31].

Table 3 presents the results of studies that examined intervention effectiveness. Among these, six studies employed a single-group pre-post design without a control group, while in the remaining studies, the control groups received a routine rehabilitation program [35], routine monitoring [40], drug treatment [5] or usual care only [7], or wait-list control [9, 38]. Various concepts were used as outcome variables to evaluate study effectiveness. In terms of physical aspects, these included functional status, fatigue, anthropometric measurements, pain, lung function, blood test results, etc. For psychosocial aspects, the variables included depression and anxiety, hope, resilience, health-related quality of life, and overall quality of life. Spiritual variables such as dignity and spiritual well-being were also used as outcome variables. The most frequently measured variables for the people with ALS/MND were quality of life (*n* = 11), followed by depression and anxiety (*n* = 8), and functional status (*n* = 5). For family members, the most frequently measured variables included depression and anxiety (*n* = 7), caregiver burden (*n* = 6), and quality of life (*n* = 4).

**Table 3:** Effects of psychoeducational interventions of included studies

Authors (year)	Contents	Control	Outcome variables (measurement instrument)	Key findings
Aoun et al. (2015) [23]	Dignity therapy	N/A	• Patients: dignity related distress (PDI), QOL (ALSAQ-5), hopefulness (HHI), spiritual well-being (FACIT-sp) • Caregivers: caregiver burden (ZBI), hopefulness (HHI), anxiety and depression (HADS)	• There were no significant differences in all outcome measures for both groups
Averill et al. (2013) [24]	Expressive disclosure	Blank	• Patients: Affect balance (ABS), depression (GDS), QOL (McGill QOL questionnaire), ambivalence over emotional expression (AEE questionnaire), emotional coping (Emotional approach coping), social constrains (social constraints scale)	• The intervention group had higher well-being than the control group at three months post-intervention, but not six months • AEE moderated three-month post-intervention well-being. Those low in AEE had higher well-being than those high in AEE regardless of condition
Bentley et al. (2014) [8	Dignity therapy	N/A	• Caregivers: Caregiver burden (ZBI), HHI, anxiety and depression (HADS)	• After controlling for the pre-post decrease in physical functioning of the person with MND, the pre-post increase in carer burden was no longer significant • There were no significant pre-test post-test changes for hopefulness, spirituality or dignity on the group level, but there were changes in hopefulness at the individual level
Bentley et al. (2014) [25]	Dignity therapy	N/A	• Patients: Hopefulness (HHI), dignity (PDI), spiritual well-being (FACIT-sp)	• There were no significant pre-test post-test changes for hopefulness, spirituality or dignity on the group level, but there were changes in hopefulness at the individual level
De Marchi et al. (2022) [34]	Chatbot webapp (dietary monitoring and nutritional recommendation)	Standard counseling (historical control population)	• Patients: body weight, Physical function (ALSFRS-R), QOL (ALSAQ-40)	• Regarding the change in weight in the Chatbot group, we observed a weight stabilization over the telehealth compared to the control group • No significant reduction in the slowdown of ALSFRS-R (both groups) • Significant increase of three subscales of ALSAQ-40 (Chatbot group)
De Wit et al. (2020) [9]	Acceptance and commitment therapy	A wait-list control group	• Caregivers: anxiety and depression (HADS), caregiver burden (ZBI), QOL (CarerQoL), • Patients: QOL (MQOL), anxiety and depression (HADS)	• The support program had no effect on psychological distress (both caregivers and patients), but may be beneficial by increasing feeling of control over the caregiving situation
Díaz et al. (2016) [5]	Cognitive behavioral therapy combined with counselling techniques	Individualized psychoactive drug treatment	• Caregivers: anxiety and depression (HADS)	• The psychological intervention demonstrated potential for the reduction of anxiety and depression levels
Fateh et al. (2022) [35]	Energy conservation program	Routine rehabilitation programs	• Patients: Fatigue (FSS), QOL (SF-36), occupational performance (COPM)	• Using energy conservation strategies could lead to better mid-term fatigue management and occupational performance improvement, but it did not improve QOL in patients with MND
Horne-Thompson & Bolger (2010) [22]	A live music therapy session, recorded music	Reading or watching TV	• Patients: Anxiety and depression (HADS), anxiety (ESAS), heart rate and oxygen saturation	• Not significant in either the music therapy or recorded music groups. The majority of participants reported little or no anxiety prior to the interventions, and therefore, little change was noted in any of the groups
Kavanaugh et al. (2020) [32]	Skills training and support program	N/A	• Caregivers: Caregiving tasks & self-efficacy (MACA-YC18), Goal attainment/behavior (5-Likert), Well-being behaviors (4-Likert), Social support (developed for the study)	• Participants reported significant increase in confidence in tasks, including communication systems and respiratory equipment Participants identified goal setting and creating behaviors to reach those goals
Kleinbub et al. (2015) [28]	A hypnosis treatment and self-hypnosis training	blank	• Patients: anxiety and depression (HADS), QOL (ALSSQOL-R), physical function (ALSFRS-R) • Caregivers: anxiety and depression (HADS)	• The statistical analyses revealed an improvement in psychological variables' scores immediately after the treatment. Amelioration in patients' and caregivers' anxiety as well as caregivers' depression, were found to persist at 3 and 6 months follow-ups • Treated patients decline in ALSFRS-r score was observed to be slower than that of control group's patients
Kolomeytseva et al. (2022) [36]	Home-based music therapy	N/A	• Patients: Pulmonary function (pulmonary function tests: MIP, MEP, FVC, PEF), swallowing (CNS-BFS)	• The music therapy was beneficial for the participants’ bulbar and respiratory functions
Pagnini et al. (2022) [38]	An ALS-specific online Langerian mindfulness training program	Wait-list control	• Patients: QOL (ALSSQOL-R), anxiety and depression (HADS), physical function (SA-ALSFRS-R) • Caregivers: QOL (SF-36), caregiver burden (ZBI)	• The experimental group reported higher levels of QOL, lower values of depression, anxiety, and negative emotions, compared to the controls • The caregivers from the mindfulness group reported lower scores of care burden, depression, and anxiety, with higher values of energy and emotional well-being over time
Palmieri et al. (2012) [29]	Hypnosis intervention	N/A	• Patients: anxiety and depression (HADS), QOL (ALSSQOL-R, ALSAQ-5) • Caregivers: anxiety and depression (HADS)	• Significant reduction of anxiety both for patients and caregivers, and significant reduction of depression for patients group only • Significant improve of total ALSSQOR-R and several subscales. Not significant difference of ALSAQ-5
Palmieri et al. (2021) [39]	Empathy-based supportive counseling intervention	Control 1: blank; Control 2: hypnosis (previous study data)	• Patients: Anxiety and depression (HADS), QOL (ALSSQOL-R)	• Depression and anxiety remained constant at the 6-month follow-up in counseling group, but the group Χ time interaction was not significant • ALSSQOL-R showed constant improvement in counseling group, and the group Χ time interaction was significant
Raglio et al. (2016) [19]	Active music therapy	Standard care	• Patients: Physical function (ALSFRS-R), anxiety and depression (HADS), QOL (MQOL), music therapy process (MTRS)	• Both groups presented a general significant improvement over time in the psychological outcomes • AMT group maintained quality of life improvement, whereas the SC group worsened
Sharbafshaaer et al. (2022) [40]	Individual tele-consult and resilience-oriented sessions of group therapy	Routine monitoring (phone call)	• Caregivers: Caregiver burden (CBI), resilience (CD-RISC), stress (PSS)	• No significant differences were found in CBI, CD-RISC, and PSS during the 9-month observation period in the treated group compared with the control group
Ugalde et al. (2018) [33]	Therapeutic group intervention	N/A	• Caregivers: Depression, anxiety, and somatization (BSI-18), burden (Caregiver reaction assessment), problem-solving confidence (Problem-solving inventory), mindfulness (Cognitive and affective mindfulness scale-revised), preparedness (Preparedness for caregiving scale)	• No significant change in measures between pre-intervention and 6 weeks post intervention
van Groenestijn et al. (2015) [7]	CBT sessions	Usual care	• Patients: QOL (SF-36-MCS, ALSAQ-40-EF), anxiety and depression (HADS) • Caregivers: QOL (SF-36-MCS), anxiety and depression (HADS), caregiver strain (CSI)	• Patients’ ALSAQ-40-EF and caregivers’ SF-36-MCS were significantly better in CBT than usual care • CSI was significantly lower in the CBT than the usual care

*ABS* Affects balance scale, *AEE* Ambivalence over emotional expression, *ALS* Amyotrophic lateral sclerosis, *ALSAQ-5* Five-items ALS assessment questionnaire, *ALSAQ-40-EF* Emotional functioning subscale of ALSAQ-40, *ALSFRS-R ALS* functional rating scale-revised, *ALSSQOL-R* ALS-specific quality of life-revised questionnaire, *BSI-18* Brief symptom inventory, *CarerQoL* Care related-quality of life, *CBI* Caregiver burden inventory, *CBT* cognitive behavioral therapy, *CD-RISC* Connor Davidson resilience scale, *COMP* Canadian occupational performance measure, *CNS-BFS* Center for neurologic study bulbar function scale, *ESAS* Edmonton symptom assessment system, *FACIT-sp* Functional assessment of chronic illness therapy-spiritual wellbeing scale, *FSS* Fatigue severity scale, *FVC* Forced vital capacity, *GDS* Geriatric depression scale, *HADS* Hospital anxiety and depression scale, *HHI* Herth hope index, *MACA-YC18* Multidimensional assessment of caring activities, *MIP* Maximal inspiratory pressure, *MEP* Maximal expiratory pressure, *MND* motor neuron disease, *MQOL* McGill quality of life questionnaire, *MTRS* Music therapy rating scale, *PDI* Patient dignity inventory, *PEF* Peak expiratory flow, *PSS* Perceived stress scale, *QOL* quality of life, *ROM* Range of motion, *SA-ALSFRS-R* Self-administered ALS functional rating scale-revised, *SF-36* 36-item short form survey, *ZBI* Zarit burden interview

In terms of experimental study effectiveness, more than half (*n* = 13) of the studies demonstrated one or more statistically significant effects. However, in seven studies, the intervention did not show statistically significant effects [8, 9, 22, 23, 25, 33, 40]. When focusing on RCT studies, Averill et al. [24] found that the expressive disclosure exercise intervention resulted in higher well-being for the intervention group compared to the control group at three-months post-intervention, but not at six months. In De Wit's study [9], Acceptance and Commitment Therapy did not reduce distress in patients and caregivers, but it appeared to empower caregivers by enhancing their sense of control in challenging situations. Fateh et al. [35] reported that an energy conservative program led to short- and mid-term improvements in fatigue and occupational performance, but did not enhance quality of life. Pagnini et al. [38] observed that participants who engaged in the online mindfulness intervention experienced improvements in their quality of life and psychological well-being compared to the control group. Raglio et al. [19] found that the active music therapy group showed significant improvements in global scores on the McGill Quality of Life questionnaire, while van Groenestijin et al. [7] identified a significant intervention effect on the mental quality of life of patients and caregivers as well as caregiver burden.

In qualitative research, the majority of studies analyzed needs of people with ALS/MND and their families during the disease course [18, 30], explored the positive and negative aspects of interventions [18, 30], and examined a range of effects in various domains. These effects encompassed improvements in mental and physical well-being [10], heightened positive emotions, and increased self-assurance in coping strategies [31], along with greater awareness of their individual circumstances [6, 30, 31]. Notably, group interventions frequently highlighted peer interactions, resulting in the provision of emotional support [6, 18, 30, 33].

## Discussion

The objectives of this study were to conduct a scoping review and present a comprehensive overview of psychosocial interventions designed for individuals and families affected by ALS/MND, while also pinpointing research gaps. This investigation scrutinized 25 psychosocial interventions from a total of 27 articles, aiming to discern the attributes and impacts of these interventions. To the best of our understanding, this scoping review is the inaugural attempt to comprehensively analyze psychosocial interventions offered to both people with ALS/MND and their families.

All of these studies were published after 2010 and no study before 2009, and the number of studies have been gradually increasing over time. However, the majority of the research is conducted in European and American countries, which may limit the generalization of these findings to patients and families in other regions. Due to cultural, social, and healthcare system differences, intervention effects may vary, highlighting the need for research in diverse regions and various cultural contexts.

In the categorization of psychosocial interventions, educational interventions were the most frequently implemented, which aligns with previous research among cancer survivors [41]. Particularly, ALS/MND patients and their families face various challenges due to the rarity of the disease, and providing information and education could enhance patient's self-care abilities and improve family caregiving skills [42]. While the interventions primarily targeted patients, there were also several interventions targeting both patients and families, as well as interventions focusing solely on families. Furthermore, some studies provided interventions to patients or families while simultaneously evaluating research outcomes from both patients and families. These findings are based on previous research indicating that ALS/MND is a condition where family support is essential, and family knowledge and mental health can directly impact patient health outcomes [2]. As patient and family health outcomes are interdependent, recent research in other chronic conditions [43, 44] has increasingly focused on interventions targeting both patients and families as a single unit, also known as a dyadic. Therefore, dyadic interventions for ALS/MND should be developed and implemented in further studies.

Most interventions were conducted in a face-to-face format, with limited research regarding online or smartphone-based interventions. After the COVID-19 pandemic, online interventions have become increasingly prevalent. Online interventions offer users the flexibility to engage in programs at their preferred times and locations, potentially reducing time and spatial constraints and increasing satisfaction [31]. However, some participants preferred 'hands-on' direct support and highlight technical issues associated with online-based interventions [31, 32]. Therefore, it is important to consider demographic characteristics and intervention content when choosing intervention mode. Moreover, participants in group interventions reported benefits such as sharing experiences and receiving emotional support beyond knowledge enhancement and improved mental health. Consequently, depending on the intervention content, group interventions may be considered as a method to enhance participant satisfaction and cost-effectiveness.

The heterogeneity of methodologies and characteristics among the analyzed studies posed challenges for direct comparisons, making it difficult to assess the overall effectiveness of psychosocial interventions. Most studies reported positive findings in terms of the feasibility and acceptability of interventions, and the qualitative interview mostly reported benefits of the interventions. While many studies presented positive effects of interventions quantitatively, some did not report statistically significant effects. However, one reason for the inconsistent results is the limited sample sizes. Most studies were conducted with less than 50 participants and only a few interventions targeted over 100 participants. Furthermore, high dropout rate could result in insufficient power to statistically test study hypothesis. Studies with long term follow ups (about 6 months after intervention completed) or administered online or at a distance, had dropout rates that were relatively high. Given that most studies were conducted with small sample sizes, even if there were actual effects, statistically significant results might not have been obtained. Furthermore, most studies reported only short-term effects, highlighting the importance of investigating the long-term effects of interventions.

### Research gaps

We identified several research gaps among the studies. The research designs of the included studies had some limitations, including small sample sizes, high dropout rates, non-randomized controlled trials, issues with measurement tools, and were limited to short-term outcomes. ALS/MND is not only a rare disease, but also one that affects various aspects of the body and progresses rapidly, making participant recruitment and reservation challenging. However, in order to analyze research results more rigorously, there is a need to improve research designs in terms of sample size and study design. While most studies employed validated measurements, there are some concerns. For instance, Hospital Anxiety and Depression Scale is widely used in various studies, however, its validity may be compromised when applied to ALS/MND patients, who have specific disease-related characteristics [45], or non-patient family members. This potentially could lead to a failure to detect the effects of interventions. Furthermore, the promising outcomes from most qualitative studies, including improvements in well-being and increased awareness of individual circumstances, suggest that the integration of mixed methods could enhance our understanding of interventions for ALS/MND.

Secondly, most interventions primarily targeted patients in at early or mid-stages of the disease. For families, the majority of studies focused on spouses. To account for demographic diversity and disease stage, it is essential to consider diversifying the target participant groups. Moreover, most quantitative studies concentrated on evaluating the effectiveness of interventions with respect to depression, anxiety, and quality of life. Consequently, future research endeavors should strive to comprehensively investigate a broader spectrum of outcomes.

This study had some limitations. Firstly, due to the inconsistent research designs, we could not perform a meta-analysis, making it challenging to assess the effects of interventions definitively. Secondly, our inclusion criteria were limited to studies published in English, potentially reducing the generalizability of the findings. Additionally, by focusing solely on original articles and case studies, we omitted gray literature and ongoing research, thus our analysis did not encompass all interventions for ALS/MND patients and their families.

## Conclusions

In conclusion, this review highlights the availability of a range of psychosocial programs tailored for people with ALS/MND and their families. These programs exhibit promising potential as supportive interventions throughout the ALS/MND care journey. While the definitive effectiveness of psychosocial interventions remains somewhat unclear in this study, a significant body of research reports favorable outcomes. Furthermore, the majority of these interventions are deemed feasible and applicable. These findings can serve as valuable guidance for clinicians, professionals, and policymakers involved in crafting and implementing interventions for individuals with ALS/MND and their families. Given the progressive and debilitating nature of this condition, coupled with the absence of a cure, the adoption of a psychosocial approach can prove beneficial for both ALS/MND patients and their families. Providing thorough education and counseling at ALS/MND clinics, as well as fostering connections with other patients' families, can positively impact the health outcomes of both patients and their families. Additionally, while there is limited research in this review, various behavioral therapies such as expressive therapy, music therapy, and psychological treatments including hypnotherapy and mindfulness are showing promising results. Therefore, further research is needed, but these interventions are worth actively considering for implementation in ALS/MND clinics.

As mentioned earlier in this review, it is challenging to draw definitive conclusions about the effectiveness of psychosocial interventions applied to ALS/MND patients and their families. Specifically, high-quality RCTs with a large sample size are recommended to examine and confirm intervention effectiveness. Due to the observed high dropout rate in this study, it is necessary to increase the sample size by recruiting participants from multiple sites. Simultaneously, actively utilizing human resources is essential when implementing online interventions to minimize research participant attrition. The integration of mixed methods research may offer valuable insights into the multifaceted nature of intervention strategies. Additionally, there is a pressing need to diversify intervention delivery methods, target populations, and content to better cater to the unique needs of individuals affected by ALS/MND.

### Supplementary Information

**Additional file 1. **Preferred Reporting Items for Systematic reviews and Meta-Analyses extension for Scoping Reviews (PRISMA-ScR) Checklist.

**Additional file 2. **Documenting the search.

## Data Availability

All the data are available from the corresponding author up on a reasonable request.

## Abbreviations

ALS/MND: Amyotrophic lateral sclerosis/motor neuron disease
CINAHL: Cumulative Index of Nursing and Allied Health
PRISMA-ScR: Preferred Reporting Items for Systematic Reviews and Meta-analyses
RCT: Randomized controlled trial
USA: United States of America
