# Supplementary material for: Psychosocial interventions for people with amyotrophic lateral sclerosis and motor neuron disease and their caregivers: a scoping review
Source: BMC Nurs. 2024 Jan 29;23:75. doi: 10.1186/s12912-024-01721-6 (PMC10823680; doi:10.1186/s12912-024-01721-6)
Supplement: Supplementary file 2 — Additional file 2. Documenting the search. [file 12912_2024_1721_MOESM2_ESM.docx]

| Additional file 1. Documenting the search | | | |
| --- | --- | --- | --- |
| Name of the database | | PubMed | |
| Dates of the search | | January 30 2023 | |
| No. | Search Query | | Results |
| #1 | "Amyotrophic Lateral Sclerosis"[Mesh] | | 22,802 |
| #2 | "Amyotrophic Lateral Sclerosis"[TW] OR Gehrig*[TW] OR "Lou Gehrig*"[TW] OR "ALS"[TW] | | 39,542 |
| #3 | #1 OR #2 | | 39,542 |
| #4 | "Psychotherapy"[Mesh] OR "Social Support"[Mesh] OR "Psychosocial Support Systems"[Mesh] OR "Patient Care Team"[Mesh] OR "Adaptation, Psychological"[Mesh] OR "Patient Education as Topic"[Mesh] | | 544,508 |
| #5 | Psychotherap*[TW] OR "Behavior Therap*"[TW] OR Psychoeducation*[TW] OR "Social Care"[TW] OR "Social Support*"[TW] OR "Psychosocial Support*"[TW] OR ((Patient*[TW] OR Multidisciplinary[TW] OR Interdisciplinary[TW]) AND ("Health Team*"[TW] OR "Health Care Team*"[TW] OR "Healthcare Team*"[TW] OR "care team*"[TW])) OR ((psycho*[TW] OR emotion*[TW] OR personal[TW]) AND (adjustment*[TW] OR adaptati*[TW])) OR (Coping[TW] AND (Behavi*[TW] OR skill*[TW] OR strateg*[TW])) OR "Patient Education*"[TW] | | 589,955 |
| #6 | #4 OR #5 | | 713,826 |
| #7 | #3 AND #6 | | 671 |
| #8 | #7 AND (("2000/01/01"[PDAT] : "3000/12/31"[PDAT]) AND (English[lang]) | | 520 |

| Name of the database | | Cochrane | | | |
| --- | --- | --- | --- | --- | --- |
| Dates of the search | | January 30 2023 | | | |
| No. | Search Query | | | Results | |
| #1 | 'amyotrophic lateral sclerosis'/exp | | | 46,711 | |
| #2 | ('Amyotrophic Lateral Sclerosis' OR Gehrig* OR 'Lou Gehrig*' OR 'ALS'):ab,ti,kw | | | 56,939 | |
| #3 | #1 OR #2 | | | 67,277 | |
| #4 | 'psychotherapy'/exp OR 'social support'/exp OR 'psychosocial care'/exp OR 'patient care'/exp OR 'psychological adjustment'/exp OR 'patient education'/exp | | | 1,481,775 | |
| #5 | (Psychotherap* OR 'Behavior Therap*' OR Psychoeducation* OR 'Social Care' OR 'Social Support*' OR 'Psychosocial Support*' OR ((Patient* OR Multidisciplinary OR Interdisciplinary) NEAR/3 ('Health Team*' OR 'Health Care Team*' OR 'Healthcare Team*' OR 'care team*')) OR ((psycho* OR emotion* OR personal) NEAR/3 (adjustment* OR adaptati*)) OR (Coping NEAR/3 (Behavi* OR skill* OR strateg*)) OR 'Patient Education*'):ab,ti,kw | | | 258,867 | |
| #6 | #4 OR #5 | | | 1,571,776 | |
| #7 | #3 AND #6 | | | 2,379 | |
| #8 | #17 AND ([article]/lim OR [article in press]/lim OR [review]/lim) AND ([english]/lim) AND [2000-2023]/py | | | 1,232 | |
| Name of the database | | EMBASE | | |  |
| Dates of the search | | January 30 2023 | | |  |
| No. | Search Query | | Results | |  |
| #1 | [mh "Amyotrophic Lateral Sclerosis"] | | 656 | |  |
| #2 | ("Amyotrophic Lateral Sclerosis" OR Gehrig* OR "Lou Gehrig*" OR "ALS"):ab,ti,kw | | 2,480 | |  |
| #3 | #1 OR #2 | | 2,480 | |  |
| #4 | [mh "Psychotherapy"] OR [mh "Social Support"] OR [mh "Psychosocial Support Systems"] OR [mh "Patient Care Team"] OR [mh "Adaptation, Psychological"] OR [mh "Patient Education as Topic"] | | 41,722 | |  |
| #5 | (Psychotherap* OR "Behavior Therap*" OR Psychoeducation* OR "Social Care" OR "Social Support*" OR "Psychosocial Support*" OR ((Patient* OR Multidisciplinary OR Interdisciplinary) NEAR/3 ("Health Team*" OR "Health Care Team*" OR "Healthcare Team*" OR "care team*")) OR ((psycho* OR emotion* OR personal) NEAR/3 (adjustment* OR adaptati*)) OR (Coping NEAR/3 (Behavi* OR skill* OR strateg*)) OR "Patient Education*"):ab,ti,kw | | 51,831 | |  |
| #6 | #4 OR #5 | | 69,062 | |  |
| #7 | #3 AND #6 | | 75 | |  |

| Name of the database | | CINAHL | |
| --- | --- | --- | --- |
| Dates of the search | | January 30 2023 | |
| No. | Search Query | | Results |
| #1 | (MH "Amyotrophic Lateral Sclerosis") | | 4,731 |
| #2 | "Amyotrophic Lateral Sclerosis" OR Gehrig* OR "Lou Gehrig*" OR "ALS" | | 11,148 |
| #3 | S1 OR S2 | | 11,148 |
| #4 | (MH "Psychotherapy+") OR (MH "Support, Social+") OR (MH "Support, Psychosocial+") OR (MH "Multidisciplinary Care Team+") OR (MH "Adaptation, Psychological+") OR (MH "Patient Education+") | | 384,522 |
| #5 | Psychotherap* OR "Behavior Therap*" OR Psychoeducation* OR "Social Care" OR "Social Support*" OR "Psychosocial Support*" OR ((Patient* OR Multidisciplinary OR Interdisciplinary) N3 ("Health Team*" OR "Health Care Team*" OR "Healthcare Team*" OR "care team*")) OR ((psycho* OR emotion* OR personal) N3 (adjustment* OR adaptati*)) OR (Coping N3 (Behavi* OR skill* OR strateg*)) OR "Patient Education*" | | 310,556 |
| #6 | S4 OR S5 | | 448,921 |
| #7 | S3 AND S6 | | 923 |
| #8 | S7 (Limit to date 2000-) | | 698 |
